# Multiple Traditional Chinese Medicine interventions for idiopathic pulmonary fibrosis: A protocol for systematic review and meta-analysis of overview

**DOI:** 10.1097/MD.0000000000022396

**Published:** 2020-09-25

**Authors:** Hao-Yang Zhang, Li-Jian Pang, Xiao-Dong Lv, Chuang Liu, Ming-Hua Nan

**Affiliations:** a Graduate School, Liaoning University of Traditional Chinese Medicine; b Respiratory department, Affiliated Hospital of Liaoning University of Traditional Chinese Medicine; c Liaoning University of Traditional Chinese Medicine; d Emergency Department, Affiliated Hospital of Liaoning University of Traditional Chinese Medicine; e Cardiovascular department, The Second Affiliated Hospital of Liaoning University of Traditional Chinese Medicine, Shenyang, Liaoning, China.

**Keywords:** idiopathic pulmonary fibrosis, overview, protocol, systematic review, traditional chinese medicine

## Abstract

**Background:**

The therapeutic strategies of idiopathic pulmonary fibrosis (IPF) tend to be comprehensive. Improving the major symptoms and quality of life (QoL) is as important as postponing the process of fibrosis. However, only pirfenidone and nintedanib conditionally recommended by guidelines and no definite proof indicate that they can significantly ameliorate the main symptoms and QoL of IPF sufferers. At present, multiple types of Traditional Chinese Medicine (TCM) interventions alone or in combination with conventional western medicine managements are widespreadly applied in IPF treatment, which seemingly have a promising clinical effect, especially in ameliorating the main symptoms and improving QoL. Subsequently, the number of relevant studies in systematic reviews(SRs) and meta-analyses of randomized controlled trials(RCTs) increased significantly. Hence, we plan to implement an overview to collect, evaluate, and summarize the results of these SRs.

**Methods:**

An all-round literature retrieval will be conducted in 9 electronic databases, including PubMed, EMBASE, CINAHL, Cochrane Library, Epistemonikos, CNKI, CBM, Wanfang, and VIP. We will focus on the systematic review and meta-analysis of RCTs for multiple TCM interventions alone or in combination with routine western medicine measures in IPF treatment. The main outcomes we follow with interest include the improvement of major symptoms (cough, dyspnea) and QoL. Secondary outcomes will consist of minor symptoms improvement, clinical total effective rate, lung function, blood gas analysis, 6-minute walk text, adverse events, acute exacerbation, all-cause mortality, and IPF-related mortality. Two reviewers will independently select the SRs satisfactory with the enrolling criteria, extract key characteristics, and datas on predefined form, evaluate methodological quality by AMSTAR-2, ROBIS and PRISMA tools, and the quality of evidences adopting GRADE method. In case of any divergence will be reached an agreement by discussion or adjudicated by a third senior reviewer. We will perform a narrative synthesis of the proofs from SRs included.

**Results:**

The findings of this overvew will be presented at relevant conferences and submitted for peer-review publication.

**Conclusions:**

We expect to obtain comprehensive and reliable evidence of IPF treated by diversified TCM interventions from the potential standard SRs, which may provide suggestions for future RCTs and SRs.

**Registration number:**

INPLASY 202080110

## 1 Introduction

Idiopathic pulmonary fibrosis (IPF) is a chronic, progressive and fibrotic interstitial lung disease (ILD) characterized by extensive pulmonary remodeling caused by abnormal deposition of extracellular matrix.^[1]^ The etiology of IPF is still unclear, and it often occurs in middle-aged and elderly people.^[2]^ IPF is the most common idiopathic interstitial pneumonia (IIP), accounting for about 60% of cases.^[3]^

It is estimated that the annual incidence of IPF is 6.8 to 16.3/100,000,^[4]^ and about 40,000 new cases are diagnosed each year in Europe.^[5]^ There is a lack of large-scale epidemiological study in China. Nevertheless, the ILD epidemiological document indicates that its morbidity in China have an increasing tendency, what's more, with the surging of domestic population aging, the number of patients will show continuously increasing trend accompanied by growth of disease burden.^[6]^ IPF has a poor prognosis, with a median survival of 2 to 3 years after diagnosis.^[1,2]^ The recent study suggests that IPF survival has not improved significantly and mortality seems to be rising, although this may partly reflect improved recognition and diagnosis.^[7]^

The pathogenesis of IPF is complex and complicated, but there are sufficient proofs bear out that its mechanism is closely related to immunity and inflammation, involving a variety of cytokines and signaling pathways.^[8]^ Transforming growth factor-β (TGF-β) is considered to be probably the principal profibrotic cytokine within them.^[9]^ Additionally, connective tissue growth factor, platelet-derived growth factor, vascular endothelial growth factor, interleukin-1α, tumor necrosis factor-α, and interferon-γ are closely related to IPF development. The involved pathways mainly include wnt/β-catenin, shh (sonic hedgehog) and notch signaling pathway and so on.^[10]^ Recent literatures suggest that epigenetic regulation mechanism may be interrelated with occurrence and development of the disease, in which the DNA methylation, histone modification and micro RNA changes are probably the key factors in triggering IPF.^[11]^

Dry cough and dyspnea are the main clinical manifestations of IPF, within which the nonproductive cough is an irritating symptom presenting in 73% to 86% of patients.^[12]^ Besides, breathless has been proven to be bound up with survival and tend to impaired lung function as the condition progresses.^[13]^ Owing to progressive worsening of symptoms and irreversible deterioration in lung function, patients will become more debilitated and progressively restricted in activity, which probably bring about a lower quality of life (QoL).^[14]^

Therefore, the therapeutic strategies of IPF should be comprehensive. Alleviating symptoms, improving QoL, and postponing disease progression are of equal importance for cases.^[15]^ However, currently available options for conventional drugs in western medicine are limited, only pirfenidone and nintedanib are conditionally recommended by evidence-based guidelines for IPF therapy.^[16]^ Pirfenidone is an oral multi-target small molecule therapeutic drug with effects of anti-inflammatory, antioxidant, and anti-fibrotic.^[17]^ Nintedanib, a small molecule tyrosine kinase inhibitor (TKI), plays an antifibrotic role by blocking intracellular signal transduction, fibroblast proliferation, migration, and transformation.^[18]^ However, neither drug can take a turn for the worse in fibrosis progression,^[15–17]^ mild to moderate adverse events such as gastrointestinal symptoms and abnormal liver function often occur during the application of both drugs.^[19,20]^ Furthermore, there are insufficient proofs corroborate that the 2 medications can significantly improve the major symptoms and QoL of sufferers, and the high price seriously hinders the application of patients in China.^[6]^

Therefore, some deficiencies and gaps need to be further filled. Traditional Chinese Medicine (TCM) has been used for thousands of years to treat respiratory diseases in China and some other countries in Asia. Although TCM is not the mainstream treatment for IPF, it has been increasingly accepted as a form of complementary and alternative medicine in western countries.^[21]^ There are many types of TCM interventions, including Chinese herb formulas (CHFs), acupuncture, moxibustion, acupoint application, and so on. They have been well authenticated that many CHFs or extracts possess the effects on regulating cytokines, signal transduction pathways, and oxidative stress, as well as inhibiting extracellular matrix synthesis.^[22–24]^ What is more exhilarating is that the latest researches provide additional evidence that some CHFs are inclined to affect epigenetic mechanism to achieve the therapeutic purpose.^[25]^ Due to the diversity of active ingredients in CHF compositions and the potential synergistic effect among them, which enable them to have a wide-ranging targets and multiple therapeutic mechanisms.^[26]^ Moreover, acupuncture, moxibustion, and acupoint application also have the functions of regulating and improving inflammation and immunity.^[27–29]^

In recent years, a great quantity of clinical trials has been carried out in the treatment of IPF by multiple types of TCM interventions alone or in combination with conventional western medicine measures. Many trials have shown that these TCM interventions seem to have positive significance for IPF treatment, especially for the improvement of symptoms and QoL.^[26]^ Subsequently, the number of SRs and meta-analyses pooling these results increased significantly.^[30–32]^ Currently, there is still no overview of systematic review (OoSR) to synthesize the evidences of effectiveness of multiple TCM interventions, either alone or combined with routine western medicine measures, on the main symptoms and QoL of IPF patients in these SRs. Only 1 OoSR protocol has been published, which mainly focuses on CHFs as intervention in the therapy of pulmonary fibrosis, and the indicator of symptoms improvement is limited to TCM symptom score.^[33]^ Consequently, for the sake of systematically collecting, evaluating, and summarizing the proofs in these SRs, we project to perform an OoSR and drafted this protocol. This OoSR will officially assess the methodological quality of SRs included and the certainty of evidence in SRs, and we will implement a narrative synthesis of the evidence-based of our interest from the selected SRs.

## 2 Objective

The purpose of this OoSR is to summarize SRs and meta-analyses that assess the effects of multiple TCM interventions alone or combined with conventional western medicine treatment measures for the improvement of main symptoms and QoL in IPF patients. We will include potential proofs pooled in all relevant SRs, present the latest evidence body, and report our findings in a descriptive way.

## 3 Methods

### 3.1 Study protocol and registration

This protocol was designed in accordance with Preferred Reporting Items for Systematic Reviews and Meta-Analyses Protocols (PRISMA-P) 2015 checklist.^[34]^ It is registered on the International Platform of Registered Systematic Review and Meta-analysis Protocols (INPLASY no. 202080110, https://inplasy.com/)

### 3.2 Eligibility criteria

#### 3.2.1 Types of reviews

We will include published and peer-reviewed SRs based on RCTs, and provide meta-estimates of the indicators of main symptoms (cough, dyspnea) and/or QoL. SRs published only in abstract, without meta-analyses, non-SRs, or other overviews will be excluded. We will not be place restrictions on publication time of SRs and RCTs included.

#### 3.2.2 Participants

We will restrict our overview to studies of human patients with IPF in stable stage. We will exclude meta-analyses of trials exclusively populations in IPF with acute exacerbation or IPF in stable period with other respiratory diseases.

#### 3.2.3 Interventions

TCM therapy alone or combined with routine western medicine measures should be applied in the treatment group. TCM interventions comprise CHFs, acupuncture, moxibustion, and acupoint application.

#### 3.2.4 Comparisons

Intervention measures in the control group we defined consist of conventional pharmacotherapy, placebo, oxygen therapy, and no treatment.

#### 3.2.5 Outcomes

##### 3.2.5.1 Primary outcomes

We are interested in indicators of major symptoms (dry cough, dyspnea) and QoL improvement, and therefore all reliable measurements of them will meet the criteria, such as TCM symptom score (dry cough, dyspnea), the St.George's respiratory questionnaire, Leicester cough questionnaire, the breathing problems questionnaire, the MOS item short from health survey, a tool to asses QoL in IPF, and so on.

##### 3.2.5.2 Secondary outcomes

The types of secondary outcome measurements contain improvement of minor symptoms, total clinical effective rate, pulmonary function, blood gas analysis, 6-minute walking test, adverse events, acute exacerbation, all-cause mortality, IPF-related mortality.

### 3.3 Information sources

An overall retrieval will be performed in the following digital databases: PubMed, EMBASE, CINAHL (Cumulative Index to Nursing and Allied Health Literature), Cochrane Library and Epistemonikos, China National Knowledge Infrastructure, Chinese Biomedical Literature Database, WangFang Database, and Chinese Scientific Journal Database. The range of search time is from their inceptions to June 2020. The language of published SRs will be restricted in English and Chinese. The bibliographies of identified articles and gray literature will also be searched.

We will seek help from experts in the IPF domain to confirm other potential SRs. A medical librarian will establish and run a retrieval formula to identify relevant studies. The search strategy will undergo internal peer review. The document retrieval strategy in PubMed is as follows and we will adapt it for each database.

**Figure:**
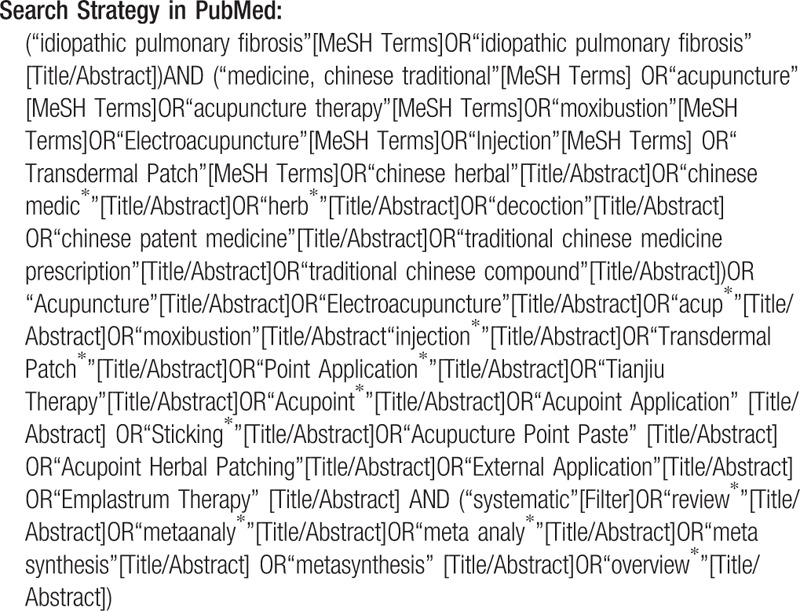


### 3.4 Studies selection and data collection process

The selection of SRs and also the extraction of key characteristics will progress in duplicate. We will use NoteExpress V3.0 software to manage retrieved studies and eliminate duplications. First, 2 investigators (ZHY and PLJ) will independently and separately screen the titles and abstracts, and further select eligible literatures by reading the full texts. The consistency of screening and selection will be measured by Kappa statistics, and any disagreements will be resolved by discussion or arbitration by a third senior reviewer (LXD). The reviewers will document all studies that do not meet the criteria and provide reasonable reasons for exclusion in the process. Inconformity studies with justifications will also be reported in the table.

Two reviewers (ZHY and LC) will independently extract key characteristics and data from all standard-compliant systematic reviews and meta-analyses, and use predefined form designed to summarize the informations of each SR. Any divergence will be adopted by discussion or adjudicated by a third senior reviewer (LXD). If any important information elements are missing, inadequate or inconsistent reporting in SRs, we will attempt to contact the authors for desired data or obtain directly from the original study.

The reviewers will collect following data items from each SR included: basic informations of SR (title, author, country, publication time, funding, conflicts of interest); basic informations of primary study (author, country, publication time, study design); search strategy (database, time range, retrieval date); population characteristics (age, sex, race, course of disease, diagnostic criteria, setting); interventions in treatment group (type, dosage form, dose, intensity, frequency, course of treatment); interventions in control group (type, dosage form, dose, intensity, frequency, course of treatment); primary and secondary outcome measures; results (risk of bias in original studies, total number of studies and cases, meta-estimate, detection and reporting of subgroups).

### 3.5 Assessment of methodological quality of included reviews

Assessment of multiple systematic reviews-2 (AMSTAR-2) is a tool used to measure the methodological quality, which has been demonstrated relatively simple, reliable, and effective for methodological evaluation of SRs.^[35]^ The AMSTAR-2 tool comprises 16 items, covering the whole process of SR in topic selection, design, registration, information extraction, data statistical analysis, and discussion. According to the guidance document of AMSTAR-2, the overall methodological quality of each SR may be classified as “high,” “moderate,” “low,” and “very low.” Scores will be completed and calculated through the online AMSTAR-2 checklist (https://amstar.ca/Amstar_Checklist.php) in this study.

Risk of bias in systematic reviews (ROBIS) is a new tool for assessing the bias risk in the design, execution, and analysis processes of SRs,^[36]^ which mainly consist of 3 phases: evaluating the goodness of fit between the issue to be solved in SRs and the target one, identifying the extent of risk of bias in production process, and judging the overall bias risk of SRs. The responses to each iconic question in ROBIS tool mainly including “yes” “probably yes” “no”“probably no” and “no information.”

We will also appraise reporting quality of each SR included by referring to criteria specified in Preferred Reporting Items for Systematic Reviews and Meta-Analyses (PRISMA),^[37]^ and further embody the integrity and transparency of this overview.

The quality assessment of methodology will be separately completed by 2 reviewers (ZHY and LC), and in case of any disagreements in the process will be settled through discussion or judged by a third senior reviewer (LXD) if necessary. We will calculate kappa statistics to understand the consistency of assessments by AMSTAR-2, ROBIS, and PRISMA between 2 reviewers. Kappa <0.2 is considered as “poor agreement,” 0.2 to 0.4 as “fair agreement,” 0.4 to 0.6 as “moderate agreement,” 0.6 to 0.8 as “substantial agreement,” and 0.8 to 1.0 as “almost perfect agreement.” Based on the evaluation results, we will not exclude any SR during this phase. This overview will not reassess the risk of bias of primary studies in SRs included; instead, we will collect their results of individual study as well as evaluating methods, and report any findings in the review.

### 3.6 Assessment of quality of evidence in included reviews

The quality of evidence pooled within the SRs and meta-analyses will be assessed independently by 2 overview authors (ZHY and NMH) using Grading of Recommendations Assessment, Development and Evaluation (GRADE) method, which is an algorithm developed to assign levels of meta-analysis evidence for OoSR study types.^[38]^ According to this approach, RCTs begin to be identified as high-quality evidence to support the estimation of intervention effects. Five factors including study limitations/risk of bias, publication bias, imprecision, inconsistency and indirectness that may lead to reduce the level of evidence, and 3 factors including large magnitude of an effect, dose–response gradient, and effect of plausible residual confounding tend to increase the quality of evidence. The overall quality of proof will be judged as “high,” “moderate,” “low,” or “very low”. Differences over the rating quality of evidence will be reached an agreement through consultation or adjudication by a third senior researcher (LXD).

### 3.7 Data synthesis

This overview is designed to collect and present the current evidence body of improvement in symptoms and QoL of IPF treated with various TCM interventions alone or combined conventional western medicine measures, and data from primary studies may be pooled to estimate for several times in relevant SRs. Thus, this study will not consider the overlap of original studies between SRs, and we will not perform meta-analysis. However, we will assess it to understand the overall extent of coverage, describe the number and scale of overlapping primary studies with their weight in the analysis through a narrative way, and then create a table to visually demonstrate it.

We will generate and present a summary of the results of primary and secondary outcome measures in all included SRs. When a meta-analysis was performed, we will report the relative risk, odds ratio or hazard ratio for dichotomous outcomes, and weighted mean difference or standard mean difference (SMD) for continuous outcomes with their 95% confidence intervals and heterogeneity estimates.

We will employ the PRISMA flow chart to summarize the screening process of the studies, report the key characteristics extracted from the SRs using a predesigned table, present the results of methodological quality assessed by AMSTAR-2, ROBIS, and PRISMA in tabular form, demonstrate the pool effect estimates with their confidence of evidence adopting forms and forest plots.

### 3.8 Strengths and limitations of this study

1. This study will be the first OoSR to integrate and summarize the relevant evidence of treating IPF with TCM intervention measures.
2. We will use multiple tools to formally assess the quality of methodology and evidence included in the SRs to reflect the integrity and transparency of this article.
3. The language of literature retrieval is limited to Chinese and English, which may lead to omission.

## 4 Discussion

We intend to carry out a formal OoSR and have drafted this manuscript of protocol. The overview will be summarized evidence based on the effectiveness of multiple TCM interventions alone or in combination with conventional western medicine measures to alleviate the main symptoms and QoL of patients with IPF; in addition, other proofs of efficacy and safety will also be generalized. This study will systematically collect, evaluate, and synthesize these results, which may benefit clinicians, policy deciders, and clinical guideline makers. We expect that the results of this overview will highlight the gaps in current evidence, which will provide suggestions for future RCTs and SRs.

## Author contributions

**Conceptualization:** Hao-yang Zhang, Xiao-Dong Lv, Li-Jian Pang.

**Investigation:** Hao-yang Zhang, Xiao-Dong Lv, Li-Jian Pang, Chuang Liu, Ming-Hua Nan.

**Methodology:** Hao-yang Zhang, Xiao-Dong Lv, Li-Jian Pang, Chuang Liu, Ming-Hua Nan.

**Project administration:** Hao-yang Zhang, Li-Jian Pang.

**Resources:** Ming-Hua Nan.

**Writing – original draft:** Hao-yang Zhang.

**Writing – review & editing:** Hao-yang Zhang, Xiao-Dong Lv, Li-Jian Pang, Chuang Liu, Ming-Hua Nan.

## References

[1] Raghu G Remy-Jardin M Myers JL Diagnosis of Idiopathic Pulmonary Fibrosis. An Official ATS/ERS/JRS/ALAT Clinical Practice Guideline. Am J Respir Crit Care Med 2018;198:e44–68. 30168753 10.1164/rccm.201807-1255ST

[2] Raghu G Chen SY Yeh WS Idiopathic pulmonary fibrosis in US Medicare beneficiaries aged 65 years and older: incidence, prevalence, and survival, 2001-11 [published correction appears in Lancet Respir Med. 2014;2:e12]. Lancet Respir Med 2014;2:566–72. 24875841 10.1016/S2213-2600(14)70101-8

[3] Raghu G Weycker D Edelsberg J Incidence and prevalence of idiopathic pulmonary fibrosis. Am J Respir Crit Care Med 2006;174:810–6. 16809633 10.1164/rccm.200602-163OC

[4] Nalysnyk L Cid-Ruzafa J Rotella P Incidence and prevalence of idiopathic pulmonary fibrosis: review of the literature. Eur Respir Rev 2012;21:355–61. 23204124 10.1183/09059180.00002512 PMC9487229

[5] Lynch JP 3rd Huynh RH Fishbein MC Idiopathic pulmonary fibrosis: epidemiology, clinical features, prognosis, and management. Semin Respir Crit Care Med 2016;37:331–57. 27231859 10.1055/s-0036-1582011

[6] Jianwei X Yongji L Duodong R Direct economic burden of patients with idiopathic pulmonary fibrosis in China. China Journal of Pharmaceutical Economics 2019;14:9–12.

[7] Raghu G Collard HR Egan JJ An official ATS/ERS/JRS/ALAT statement: idiopathic pulmonary fibrosis: evidence-based guidelines for diagnosis and management. Am J Respir Crit Care Med 2011;183:788–824. 21471066 10.1164/rccm.2009-040GL PMC5450933

[8] Zolak JS de Andrade JA Idiopathic pulmonary fibrosis. Immunol Allergy Clin North Am 2012;32:473–85. 23102062 10.1016/j.iac.2012.08.006

[9] Wuyts WA Agostini C Antoniou KM The pathogenesis of pulmonary fibrosis: a moving target. Eur Respir J 2013;41:1207–18. 23100500 10.1183/09031936.00073012

[10] Wolters PJ Collard HR Jones KD Pathogenesis of idiopathic pulmonary fibrosis. Annu Rev Pathol 2014;9:157–79. 24050627 10.1146/annurev-pathol-012513-104706 PMC4116429

[11] Yang IV Schwartz DA Epigenetics of idiopathic pulmonary fibrosis. Transl Res 2015;165:48–60. 24746870 10.1016/j.trsl.2014.03.011 PMC4182166

[12] Hope-Gill BD Hilldrup S Davies C A study of the cough reflex in idiopathic pulmonary fibrosis. Am J Respir Crit Care Med 2003;168:995–1002. 12917229 10.1164/rccm.200304-597OC

[13] Yount SE Beaumont JL Chen SY Health-related quality of life in patients with idiopathic pulmonary fibrosis. Lung 2016;194:227–34. 26861885 10.1007/s00408-016-9850-y

[14] Nishiyama O Taniguchi H Kondoh Y A simple assessment of dyspnoea as a prognostic indicator in idiopathic pulmonary fibrosis. Eur Respir J 2010;36:1067–72. 20413545 10.1183/09031936.00152609

[15] Belkin A Swigris JJ Health-related quality of life in idiopathic pulmonary fibrosis: where are we now? Curr Opin Pulm Med 2013;19:474–9. 23851327 10.1097/MCP.0b013e328363f479

[16] Hunninghake GM A new hope for idiopathic pulmonary fibrosis. N Engl J Med 2014;370:2142–3. 24836311 10.1056/NEJMe1403448

[17] King TE Jr Bradford WZ Castro-Bernardini S A phase 3 trial of pirfenidone in patients with idiopathic pulmonary fibrosis [published correction appears in N Engl J Med. 2014;371:1172]. N Engl J Med 2014;370:2083–92. 24836312 10.1056/NEJMoa1402582

[18] Richeldi L du Bois RM Raghu G Efficacy and safety of nintedanib in idiopathic pulmonary fibrosis [published correction appears in N Engl J Med. 2015;373:782]. N Engl J Med 2014;370:2071–82. 24836310 10.1056/NEJMoa1402584

[19] Albera C Costabel U Fagan EA Efficacy of pirfenidone in patients with idiopathic pulmonary fibrosis with more preserved lung function. Eur Respir J 2016;48:843–51. 27471208 10.1183/13993003.01966-2015

[20] Kolb M Richeldi L Behr J Nintedanib in patients with idiopathic pulmonary fibrosis and preserved lung volume. Thorax 2017;72:340–6. 27672117 10.1136/thoraxjnl-2016-208710 PMC5520269

[21] Hu J Zhang J Zhao W Cochrane systematic reviews of Chinese herbal medicines: an overview. PLoS One 2011;6:e28696. 22174870 10.1371/journal.pone.0028696 PMC3235143

[22] Kao ST Wang SD Lin CC Jin Gui Shen Qi Wan, a traditional Chinese medicine, alleviated allergic airway hypersensitivity and inflammatory cell infiltration in a chronic asthma mouse model. J Ethnopharmacol 2018;227:181–90. 30172058 10.1016/j.jep.2018.08.028

[23] Zhang ZJ Wu WY Hou JJ Active constituents and mechanisms of Respiratory Detox Shot, a traditional Chinese medicine prescription, for COVID-19 control and prevention: network-molecular docking-LC-MSE analysis. J Integr Med 2020;18:229–41. 32307268 10.1016/j.joim.2020.03.004 PMC7195604

[24] Guo JY Wang DM Wang MJ Systematically characterize the substance basis of Jinzhen oral liquid and their pharmacological mechanism using UPLC-Q-TOF/MS combined with network pharmacology analysis. J Food Drug Anal 2019;27:793–804. 31324295 10.1016/j.jfda.2019.05.007 PMC9307031

[25] Li LC Kan LD Traditional Chinese medicine for pulmonary fibrosis therapy: Progress and future prospects. J Ethnopharmacol 2017;198:45–63. 28038955 10.1016/j.jep.2016.12.042 PMC7127743

[26] Zhang S Wu H Liu J Medication regularity of pulmonary fibrosis treatment by contemporary traditional Chinese medicine experts based on data mining. J Thorac Dis 2018;10:1775–87. 29707332 10.21037/jtd.2018.03.11 PMC5906336

[27] Xu FQ Feng YY Guo L The effective method for investigation meridian tropism theory in rats. Afr J Tradit Complement Altern Med 2012;10:356–67. 24146462 10.4314/ajtcam.v10i2.23 PMC3746585

[28] Liu Y Ji B Zhao G Protective effect of electro-acupuncture at maternal different points on perinatal nicotine exposure-induced pulmonary dysplasia in offspring based on HPA axis and signal transduction pathway. Biochem Biophys Res Commun 2018;505:586–92. 30274776 10.1016/j.bbrc.2018.09.145

[29] Li J Wu S Tang H Long-term effects of acupuncture treatment on airway smooth muscle in a rat model of smoke-induced chronic obstructive pulmonary disease. Acupunct Med 2016;34:107–13. 26345700 10.1136/acupmed-2014-010674 PMC4853589

[30] Wu Q Zhou Y Feng FC Effectiveness and safety of chinese medicine for idiopathic pulmonary fibrosis: a systematic review and meta-analysis. Chin J Integr Med 2019;25:778–84. 29335860 10.1007/s11655-017-2429-5

[31] Zhang Y Gu L Xia Q Radix astragali and radix angelicae sinensis in the treatment of idiopathic pulmonary fibrosis: a systematic review and meta-analysis. Front Pharmacol 2020;11:415. 32425767 10.3389/fphar.2020.00415 PMC7203419

[32] Ji K Ma J Wang L Efficacy and safety of Traditional Chinese Medicine in idiopathic pulmonary fibrosis: a meta-analysis. Evid Based Complement Alternat Med 2020;2020:1752387. 32104189 10.1155/2020/1752387 PMC7040417

[33] Li LJ Chen X Yang WN Traditional Chinese medicine for the treatment of pulmonary fibrosis: a protocol for systematic review and meta-analysis of overview. Medicine (Baltimore) 2020;99:e21310. 32756114 10.1097/MD.0000000000021310 PMC7402754

[34] Moher D Shamseer L Clarke M Preferred reporting items for systematic review and meta-analysis protocols (PRISMA-P)2015 statement. Syst Rev 2015;4:1. 25554246 10.1186/2046-4053-4-1 PMC4320440

[35] Shea BJ Reeves BC Wells G AMSTAR 2:a critical appraisal tool for systematic reviews that include randomised or non-randomised studies of healthcare interventions, or both. BMJ 2017;358:j4008. 28935701 10.1136/bmj.j4008 PMC5833365

[36] Whiting P Savović J Higgins JP ROBIS:a new tool to assess risk of bias in systematic reviews was developed. J Clin Epidemiol 2016;69:225–34. 26092286 10.1016/j.jclinepi.2015.06.005 PMC4687950

[37] David M Alessandro L Jennifer T Entries for priority reports of systematic reviews and meta-analyses:PRISMA statement. J Chin Integr Med 2009;7:889–96.

[38] Pollock A Farmer SE Brady MC An algorithm was developed to assign grade levels of evidence to comparisons within systematic reviews. J Clin Epidemiol 2016;70:106–10. 26341023 10.1016/j.jclinepi.2015.08.013 PMC4742519

